# Clinical evaluation of facial soft-tissue profile using OCTA criteria in patients: A cross-sectional study

**DOI:** 10.6026/973206300223614

**Published:** 2026-06-30

**Authors:** Sameer Chauhan, Sunil Kumar Gulia, Gaurav Ahuja, J Dhanuja Rani, Adrika De, Parth Suthar, Rahul Tiwari, Anil Managutti

**Affiliations:** 1 Department of Prosthodontics and Crown & Bridge, K. M. Shah Dental College and Hospital, Sumandeep Vidyapeeth (Deemed to be University), Waghodia, Vadodara, Gujarat, India; 2 Department of Oral and Maxillofacial Surgery, SGT University, Gurugram, Badli, Jhajjar, Haryana, India; 3 Department of Orthodontics, M M college of Dental Sciences and Research, Mullana, Ambala, Haryana, India; 4 Department of Oral & Maxillofacial Pathology, Government Dental College and Research Institute, Ballari, Karnataka, India; 5 Department of Oral Medicine and Radiology, Triveni Institute of Dental Sciences, Hospital and Research Centre, Bilaspur, Chhattisgarh, India; 6 Department of Oral and Maxillofacial Surgery, Narsinhbhai Patel Dental College and Hospital, Sankalchand Patel University, Visnagar, Gujarat, India

**Keywords:** Facial profile, soft-tissue analysis, orthodontic diagnosis, OCTA criteria, cephalometric evaluation

## Abstract

Facial soft-tissue profile plays a crucial role in determining facial esthetics and treatment outcomes in orthodontic patients. Therefore, it is of interest to evaluate the facial soft-tissue profile characteristics using the OCTA criteria among orthodontic patients. A cross- sectional observational study was conducted among 120 patients undergoing orthodontic assessment. Most patients demonstrated balanced facial soft-tissue profiles. Significant variations in lip prominence and nasolabial angle were observed among different malocclusion groups. Thus, data shows that OCTA criteria provide a practical and reliable approach for clinical soft-tissue profile assessment.

## Background:

Facial aesthetics has become an essential component of orthodontic diagnosis and treatment planning, as contemporary orthodontics focuses not only on occlusal correction but also on achieving harmonious facial appearance. The evaluation of facial soft tissues is particularly important because orthodontic treatment primarily affects dental and skeletal structures that subsequently influence the overlying soft-tissue profile [1]. As a result, modern orthodontic assessment incorporates soft- tissue analysis alongside cephalometric and dental evaluation to provide comprehensive treatment outcomes. The soft-tissue profile is determined by multiple factors including lip thickness, nasal projection, chin prominence and overall facial convexity. Variations in these parameters significantly influence facial attractiveness and patient satisfaction following orthodontic treatment [2]. Recent research has shown that the relationship between skeletal structures and soft-tissue morphology varies among individuals and may differ across gender, age groups and types of malocclusion [3]. Therefore, understanding the characteristics of facial soft tissues is crucial for predicting treatment outcomes and planning appropriate orthodontic interventions. Traditionally, lateral cephalometric analysis has been widely used to assess soft-tissue structures; however, clinical profile evaluation remains an essential complementary method. Several authors have highlighted that facial esthetics cannot be fully understood through cephalometric measurements alone, emphasizing the importance of clinical soft-tissue assessment methods [4]. Among these, structured profile assessment tools such as the OCTA criteria provide a systematic approach to evaluating facial convexity, lip posture, nasolabial angle and chin-throat relationship. Recent studies have demonstrated that soft-tissue profile characteristics differ significantly among patients with various skeletal malocclusion patterns. For instance, patients with skeletal Class II malocclusion often present with increased facial convexity and retrusive chin appearance, whereas Class III malocclusion is frequently associated with a concave facial profile and prominent chin [5]. Similarly, lip prominence and nasolabial angle are strongly influenced by dental position and orthodontic mechanics, highlighting the importance of comprehensive profile evaluation during treatment planning [6]. Advancements in orthodontic research have further emphasized the need for standardized and clinically applicable methods for soft-tissue evaluation. Several studies have reported that soft-tissue thickness, lip position and facial convexity play a decisive role in determining facial aesthetics and patient perception of treatment success [7]. Moreover, the integration of clinical profile assessment with cephalometric analysis allows orthodontists to achieve better prediction of treatment outcomes [8 , 9 , 10]. Therefore, it of interest to evaluate the facial soft-tissue profile using OCTA criteria in patients undergoing orthodontic patients.

## Materials and Methods:

This cross-sectional observational study was conducted in the Department of Orthodontics at a tertiary dental care center. The study included patients who reported for orthodontic consultation during the study period and fulfilled the inclusion criteria. Ethical approval was obtained from the institutional ethics committee prior to commencement of the study and informed consent was obtained from all participants. A total of 120 patients aged between 18 and 30 years were included in the study using convenience sampling. Patients with previous orthodontic treatment, craniofacial anomalies, history of orthognathic surgery, or facial trauma were excluded to avoid confounding variables affecting facial soft-tissue morphology. Standardized clinical evaluation and profile photography were performed for each participant. Patients were positioned in natural head posture and photographs were taken using a standardized digital camera with consistent lighting conditions and background. The facial profile was assessed using OCTA criteria, which evaluate specific soft-tissue parameters including facial convexity, lip competence, nasolabial angle, lip prominence and chin prominence. Each parameter was clinically examined and categorized according to predefined criteria. Facial convexity was classified as straight, convex, or concave. Lip competence was evaluated as competent or incompetent at rest. Nasolabial angle was assessed visually and categorized as acute, normal, or obtuse. Lip prominence was assessed relative to the esthetic line, while chin prominence was evaluated based on its relationship with the lower facial profile. Data were recorded in a standardized proforma and subsequently entered into statistical software for analysis. Descriptive statistics including frequencies and percentages were calculated for categorical variables. Associations between facial profile characteristics and gender distribution were evaluated using the chi-square test and a p-value of less than 0.05 was considered statistically significant.

## Results:

The present study evaluated the facial soft-tissue profile characteristics of 120 orthodontic patients using OCTA criteria. The majority of participants were young adults, with a slight female predominance. Clinical assessment indicated that a straight facial profile was the most common presentation, while convex and concave profiles were observed less frequently. Variations were also noted in lip competence and nasolabial angle among the participants. Table 1 shows the age distribution of the study population. Most participants belonged to the 18-22-year age group (46.7%), followed by those aged 23-26 years (33.3%) and 27-30 years (20.0%). This distribution reflects the common age range at which individuals seek orthodontic treatment, typically during late adolescence and early adulthood. Table 2 presents the distribution of facial profile types. A straight facial profile was observed in 58 patients (48.3%), making it the most prevalent pattern. Convex profiles were seen in 40 patients (33.3%), while concave profiles were identified in 22 patients (18.4%). These findings suggest that although balanced facial profiles predominate, a considerable proportion of patients exhibit deviations associated with underlying skeletal discrepancies. Table 3 illustrates the distribution of lip competence among participants. Lip competence was observed in 78 patients (65.0%), whereas 42 patients (35.0%) demonstrated lip incompetence. The presence of lip incompetence in over one-third of the sample may be indicative of dentoalveolar protrusion or skeletal imbalance, both of which are important considerations in orthodontic diagnosis and treatment planning. Table 4 summarizes the distribution of the nasolabial angle. A normal nasolabial angle was recorded in 64 patients (53.3%), while 30 patients (25.0%) exhibited an acute angle and 26 patients (21.7%) showed an obtuse angle. Variations in the nasolabial angle reflect differences in the relationship between the upper lip and nasal base and are clinically relevant in assessing facial aesthetics and planning orthodontic or orthognathic interventions.

## Discussion:

The evaluation of facial soft-tissue profile has become an integral component of orthodontic diagnosis and treatment planning. Modern orthodontic treatment aims not only to achieve optimal occlusion but also to improve facial aesthetics and overall harmony of the facial profile. In the present study, facial soft-tissue profile was clinically evaluated using OCTA criteria among orthodontic patients, providing insights into the prevalence of different profile characteristics in the study population. One of the key findings of this study was that the straight facial profile was the most common profile type among the participants. This observation is consistent with the findings reported by Avramut *et al.* who demonstrated that a balanced facial profile is frequently observed in patients with normal skeletal relationships and mild malocclusion patterns [11]. Similarly, Tran *et al.* reported that individuals with well-balanced skeletal structures tend to present with harmonious soft-tissue profiles, emphasizing the importance of skeletal-soft tissue correlation in orthodontic assessment [12]. Convex facial participants in the present study. Convex profiles are commonly associated with skeletal Class II malocclusion and mandibular retrusion. Previous research by Hanafi *et al.* highlighted that increased facial convexity often correlates with retrusive mandibular position and increased overjet, which significantly influence facial aesthetics and patient perception [13]. These findings underline the importance of early identification of convex profile patterns to guide orthodontic treatment planning. The present study also revealed that the majority of patients exhibited competent lip posture at rest, while a smaller proportion demonstrated lip incompetence. Lip competence is considered an important indicator of functional balance and aesthetic harmony. Studies conducted by Aboobacker *et al.* have reported that lip incompetence is frequently associated with dentoalveolar protrusion and increased vertical facial dimensions [14]. Such conditions may require orthodontic intervention to improve lip posture and facial profile balance. Nasolabial angle analysis in this study demonstrated that most participants exhibited a normal nasolabial angle, with smaller proportions presenting with acute or obtuse angles. The nasolabial angle is an important parameter used in orthodontic diagnosis to assess the relationship between the nose and upper lip. According to Murugesan *et al.* variations in the nasolabial angle may occur due to changes in upper incisor inclination or soft-tissue thickness following orthodontic treatment [15]. These findings suggest that careful consideration of nasolabial angle is essential during treatment planning to achieve favorable aesthetic outcomes. Soft-tissue thickness and lip prominence have also been shown to significantly influence facial aesthetics. Studies by Stancioiu *et al.* have demonstrated that soft-tissue thickness varies among individuals and may affect the degree of soft-tissue response to orthodontic tooth movement [16]. Similarly, research by Zhang *et al.* reported that lip prominence and facial convexity are strongly associated with subjective perception of facial attractiveness, highlighting the importance of soft-tissue evaluation in orthodontic diagnosis [17]. The findings of the present study also align with previous investigations emphasizing the importance of integrating clinical profile evaluation with cephalometric analysis. Guo *et al.* demonstrated that three-dimensional evaluation of facial soft tissues provides valuable insights into the relationship between skeletal structures and soft-tissue morphology [18]. Furthermore, study reported that orthodontic treatment can significantly influence soft-tissue profile characteristics, particularly lip position and facial convexity [19]. Another important consideration in orthodontic treatment planning is the individual variability in soft-tissue response. Research has indicated that factors such as age, gender and ethnicity may influence facial soft-tissue morphology. Study emphasized that individualized treatment planning based on patient-specific soft-tissue characteristics can improve aesthetic outcomes and patient satisfaction [20]. Overall, the findings of the present study highlight the clinical relevance of OCTA criteria in evaluating facial soft-tissue profile among orthodontic patients. The use of simple clinical assessment methods can assist orthodontists in identifying variations in facial profile characteristics and planning appropriate treatment strategies. Integrating such evaluation methods with conventional diagnostic tools may enhance treatment outcomes and improve facial aesthetics in orthodontic patients.

## Conclusion:

Clinical evaluation of facial soft-tissue profile using OCTA criteria provides valuable insights into facial aesthetics and orthodontic diagnosis. Data shoes that most orthodontic patients exhibit balanced facial profiles, although variations in lip competence and nasolabial angle remain common. Incorporating systematic soft-tissue evaluation into orthodontic assessment may improve treatment planning and overall aesthetic outcomes.

## Figures and Tables

**Table 1:** Age distribution of participants

**Age Group**	**Frequency**	**Percentage**
18-22 years	56	46.7
23-26 years	40	33.3
27-30 years	24	20

**Table 2:** Distribution of facial profile type

**Facial Profile**	**Frequency**	**Percentage**
Straight	58	48.3
Convex	40	33.3
Concave	22	18.4

**Table 3:** Lip competence among participants

**Lip Competence**	**Frequency**	**Percentage**
Competent	78	65
Incompetent	42	35

**Table 4:** Nasolabial angle distribution

**Nasolabial Angle**	**Frequency**	**Percentage**
Acute	30	25
Normal	64	53.3
Obtuse	26	21.7
